# Comparative Analysis of Lipid Indices in Predicting Residual SYNTAX Score After Primary PCI in Patients with Acute Coronary Syndrome

**DOI:** 10.3390/diagnostics16152412

**Published:** 2026-07-31

**Authors:** İsmail Ünğan, Ramazan Asoğlu

**Affiliations:** Department of Cardiology, School of Medicine, Yalova University, 77200 Yalova, Türkiye; dr.asoglu@yahoo.com

**Keywords:** coronary artery disease, residual syntax score, lipid ratios, LDL/HDL-C ratio, non-HDL/HDL-C ratio

## Abstract

**Background:** Atherosclerotic cardiovascular disease remains the leading cause of ischemic heart disease, with dyslipidemia representing one of its principal modifiable risk factors. This study sought to evaluate the association between residual coronary artery disease burden and contemporary lipid indices in patients presenting with a first episode of acute coronary syndrome. **Methods:** A total of 317 patients were retrospectively evaluated between January 2022 and June 2023. Demographic features, comorbid conditions, laboratory measurements, conventional lipid parameters, contemporary lipid ratios, SYNTAX scores (SS), and residual SYNTAX scores (rSS) were recorded. Following primary percutaneous coronary intervention (PCI), patients were categorized by residual SYNTAX score (rSS) as rSS-negative (score = 0) or rSS-positive (score ≥ 1). **Results:** Of the 317 patients, 174 had an rSS > 0, with a median rSS of 6 (IQR, 3–10). Patients with rSS > 0 had lower HDL-C and higher LDL-C/HDL-C and non-HDL-C/HDL-C ratios than those with rSS = 0. In adjusted models, HDL-C was inversely associated with rSS positivity (OR per 1-mg/dL increase, 0.965; 95% CI, 0.932–0.999), whereas the LDL-C/HDL-C ratio (OR per 1-unit increase, 1.337; 95% CI, 1.021–1.752) and the non-HDL-C/HDL-C ratio (OR per 1-unit increase, 1.257; 95% CI, 1.017–1.553) were positively associated. Baseline SYNTAX score was the dominant predictor, and none of the lipid-extended models significantly improved discrimination over the reference model. **Conclusions:** HDL-C, the LDL-C/HDL-C ratio, and the non-HDL-C/HDL-C ratio were independently associated with residual SYNTAX score positivity following primary PCI in patients presenting with a first ACS. These routinely available lipid indices may provide complementary information regarding the presence of residual coronary disease in this specific patient population.

## 1. Introduction

Cardiovascular disease (CVD) remains the leading cause of mortality worldwide, with approximately half of the CVD-related deaths resulting from ischemic causes [1]. Acute coronary syndrome (ACS) represents the first clinical manifestation of ischemic heart disease (IHD) in nearly 25% of affected individuals [2]. Notably, nearly half of the individuals admitted with ACS have multivessel disease, and the presence of non-culprit lesions has been associated with myocardial ischemia and adverse long-term clinical outcomes [3]. Atherosclerosis (ATS), the principal pathological substrate underlying IHD, is a chronic inflammatory process characterized by progressive lipid deposition, endothelial injury, and fibrotic remodeling within the arterial wall. Its development is influenced by multiple factors, with advancing age, male sex, hypertension (HT), cigarette smoking, diabetes mellitus (DM), and dyslipidemia among the most established contributors [2].

Abnormal lipoprotein metabolism plays a significant role in the pathogenesis of ATS; therefore, evaluating traditional lipid parameters is crucial for cardiovascular risk assessment [4]. In recent years, lipoprotein ratios (atherogenic indices) have been proposed to improve the predictive power of traditional lipid profiles. Some lipid indices, particularly non-HDL-C and the triglycerides (TG)/HDL-C ratio, have been reported to predict cardiovascular disease risk more effectively than individual lipid parameters [5,6]. Guidelines emphasize that non-HDL-C is superior to low-density lipoprotein cholesterol (LDL-C) in predicting the risk of atherosclerotic cardiovascular disease and recommend it as a secondary therapeutic target [7,8]. In addition, the LDL/HDL-C ratio (>2.5 in all groups), known as the Castelli risk index II (CRI-II), is considered a good indicator of CVD risk [5]. The LDL/HDL-C and non-HDL/HDL-C ratios (NHHR) have also been highlighted as superior predictors of cardiovascular events compared to traditional lipid indices, particularly LDL-C [5,9].

The SYNTAX score (SS) is an angiographic tool that quantifies the complexity and extent of coronary artery disease by incorporating the anatomical characteristics of coronary lesions [10]. The residual SYNTAX score (rSS) quantifies the burden of untreated coronary atherosclerotic lesions remaining after percutaneous coronary intervention (PCI). Elevated rSS values have consistently been associated with a higher incidence of major adverse cardiovascular events and increased long-term mortality [11]. Therefore, more aggressive secondary prevention strategies are important for patients with high rSS.

Accordingly, we investigated the association between current lipid indices and residual coronary atherosclerotic burden in patients with first-presenting ACS undergoing primary PCI. Identifying lipid markers linked to residual disease may improve personalized risk assessment and guide more intensive preventive and treatment strategies.

## 2. Methods

### 2.1. Study Design and Patient Population

This retrospective, single-center study included 317 consecutive patients who presented with an initial ACS diagnosis, underwent primary PCI at Yalova Training and Research Hospital between January 2022 and June 2023, and met predefined eligibility criteria.

Individuals with a history of coronary revascularization, younger than 18 years of age, chronic liver, kidney, or heart failure, active infection, autoimmune disease, confirmed malignancy, or who did not undergo coronary angiographic evaluation during hospitalization were not eligible for inclusion in the study. Patients who had received lipid-lowering therapy before admission were also excluded.

ACS was diagnosed and classified as ST-elevation myocardial infarction (STEMI) or non-ST-elevation myocardial infarction (NSTEMI) based on characteristic ischemic symptoms, electrocardiographic findings, and elevated cardiac troponin levels. Patients with unstable angina without elevated troponin levels were excluded from the analysis.

### 2.2. Coronary Angiography and SYNTAX Score Assessment

All coronary procedures were performed in the same laboratory using the Siemens Artis Zee angiography system. Baseline and residual SYNTAX scores were assessed by two experienced interventional cardiologists using the SYNTAX Score Calculator (version 2.28). Both authors were blinded to patients’ laboratory findings, clinical outcomes, and study group assignments during assessment. Angiographic analyses were performed with joint review as needed, and any differences in interpretation were resolved by consensus. A coronary artery diameter ≥ 1.5 mm with 50% stenosis was considered a residual lesion. Residual SS was determined by assessing untreated obstructive coronary lesions after PCI. Patients were grouped into those with negative (score = 0) and positive (score ≥ 1) rSS after primary percutaneous intervention.

### 2.3. Clinical and Laboratory Data Collection

Patient demographic characteristics, including age and sex, comorbidities, and other clinical variables, were extracted from institutional medical records. Fasting venous blood samples for lipid profile analysis were routinely collected on the first morning of hospital admission after an overnight fast of at least 8 h. For all patients, blood sampling was performed within the first 24 h of hospitalization, usually after completion of primary PCI. All biochemical analyses were performed in the same institutional biochemistry laboratory under standardized conditions throughout the study period. Serum total cholesterol, HDL-C, triglycerides, and LDL-C concentrations were measured using a Roche cobas^®^ 8000 modular analyzer (c702 module; Roche Diagnostics, Mannheim, Germany). Direct enzymatic assays were performed with the Cholesterol Gen.2, HDL-Cholesterol Plus 3rd Generation, Triglycerides, and LDL-Cholesterol Gen.3 reagent kits according to the manufacturer’s instructions. Non-HDL cholesterol was calculated as total cholesterol minus HDL-C. Remnant cholesterol was calculated as total cholesterol minus HDL-C minus LDL-C. The LDL-C/HDL-C ratio, non-HDL-C/HDL-C ratio, total cholesterol/HDL-C ratio, triglyceride/HDL-C ratio, and remnant cholesterol/HDL-C ratio were calculated using their conventional formulas.

### 2.4. Ethical Approval

The study was conducted in accordance with the ethical standards of the Declaration of Helsinki and received approval from the Ethics Committee of the Yalova University Faculty of Health Sciences (Approval No. 2025/182). Because of the study’s retrospective design, informed consent was waived.

### 2.5. Statistical Analysis

Statistical analyses were conducted using R version 4.4.0 (R Foundation for Statistical Computing, Vienna, Austria). Normality of continuous variables was assessed using the Shapiro–Wilk test and visual inspection of quantile–quantile (Q–Q) plots. Variables with a normal distribution were reported as mean ± standard deviation (SD) and compared between groups using Student’s *t*-test. In contrast, skewed variables were reported as medians (interquartile ranges [IQRs]) and analyzed using the Mann–Whitney U test. Categorical data were expressed as counts and percentages and compared using the Pearson chi-square test or Fisher’s exact test, as appropriate. A two-sided *p* value < 0.05 was considered statistically significant. The primary outcome was the presence of residual coronary disease. Multivariable binary logistic regression was used to evaluate factors associated with rSS positivity. The predefined reference model included age, sex, ACS presentation (STEMI versus NSTEMI), admission glucose concentration, and baseline SYNTAX score. Admission glucose was scaled per 10 mg/dL increase. This adjustment was intended to evaluate whether the lipid parameters were associated with residual disease beyond the coronary complexity already present before PCI. HDL-C, the LDL-C/HDL-C ratio, and the non-HDL-C/HDL-C ratio were added separately to the reference model because these parameters are biologically and mathematically interrelated and their simultaneous inclusion could result in unstable or difficult-to-interpret estimates. The linearity of continuous predictors in the logit was evaluated using restricted cubic splines with three knots. No evidence of significant non-linearity was observed for baseline SYNTAX score, age, admission glucose, HDL-C, LDL-C/HDL-C ratio, or non-HDL-C/HDL-C ratio. Multicollinearity was assessed using variance inflation factors, all of which were below 1.30. Model fit was compared using the Akaike and Bayesian information criteria and likelihood ratio tests. Discrimination was assessed using the area under the receiver operating characteristic curve with 95% confidence intervals, and differences in AUC between nested models were evaluated using paired DeLong tests. Internal validation of the reference model and the model, including the non-HDL-C/HDL-C ratio, was performed using 1000 bootstrap resamples. Optimism-corrected discrimination, calibration intercept, calibration slope, maximum calibration error, and Brier score were calculated. Several sensitivity analyses were performed. First, models excluding baseline SYNTAX score were evaluated to examine associations before adjustment for initial anatomical disease burden. Second, expanded models additionally adjusted for hypertension, diabetes mellitus, and left ventricular ejection fraction. Third, to reduce information loss associated with dichotomization, rSS was categorized as 0, 1–8, and >8, and analyzed using ordinal regression. This approach was selected instead of conventional continuous-outcome regression because the rSS distribution had a marked point mass at zero and was right-skewed, whereas the categories retained clinically interpretable information on residual disease severity. The proportional-odds assumption was assessed for each predictor. Because the baseline SYNTAX score violated this assumption, partial proportional-odds models were fitted, allowing its effect to vary across the cumulative logit thresholds while retaining common effects for the remaining predictors. Nested models were compared using likelihood-ratio tests.

## 3. Results

**Figure 1:**
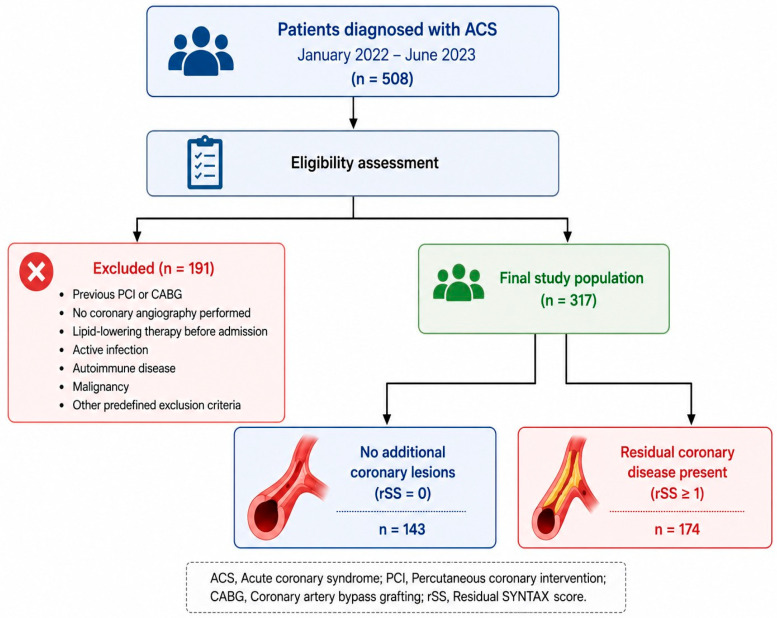
Flow diagram of patient selection and study group allocation.

A total of 317 patients were included in the analyses, of whom 143 had complete angiographic revascularization (rSS = 0), and 174 had residual coronary disease (rSS ≥ 1). The study flow, including patient selection, eligibility assessment, and final group allocation, is illustrated in Figure 1.

Baseline demographic, clinical, angiographic, and laboratory characteristics are summarized in Table 1. STEMI presentation was more common among patients with rSS = 0 than among those with rSS ≥ 1 (61.5% vs. 45.4%, *p* = 0.005). The median baseline SYNTAX score was higher in the rSS-positive group than in the rSS-negative group (12 [9–15] vs. 6 [5–7], *p* < 0.001). Among patients with rSS ≥ 1, the median residual SYNTAX score was 6 [3–10].

**Figure 2:**
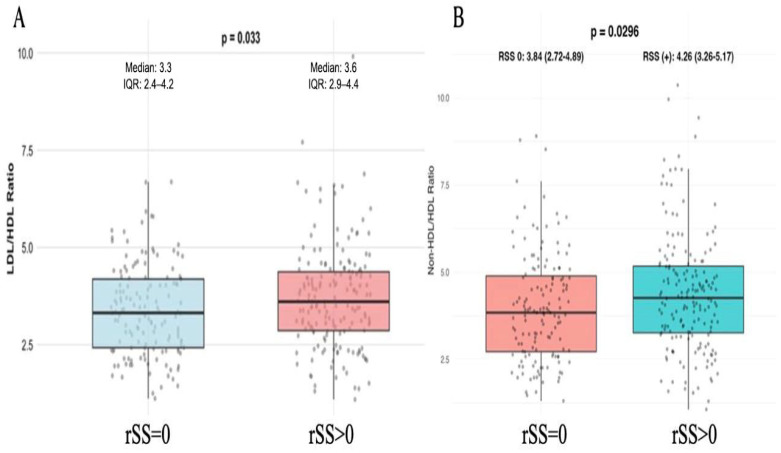
LDL-C/HDL-C ratio (**A**) and non-HDL-C/HDL-C ratio (**B**) in patients with complete angiographic revascularization (rSS = 0) and residual coronary disease (rSS > 0).

Conventional lipid parameters, including total cholesterol, triglycerides, LDL-C, remnant cholesterol, and non-HDL-C, did not differ significantly between the groups. In contrast, HDL-C was lower in patients with rSS ≥ 1 than in those with rSS = 0 (37 [32.3–43.8] vs. 39 [33–49] mg/dL, *p* = 0.022). The LDL-C/HDL-C ratio was higher in the rSS-positive group (3.6 [2.9–4.4] vs. 3.3 [2.4–4.2], *p* = 0.033), as was the non-HDL-C/HDL-C ratio (4.3 [3.3–5.2] vs. 3.8 [2.7–4.9], *p* = 0.030). The TG/HDL-C ratio and the evaluated inflammatory cell/HDL-C indices did not differ significantly between the groups. The distributions of the LDL-C/HDL-C and non-HDL-C/HDL-C ratios are shown in Figure 2.

In the reference logistic regression model, baseline SYNTAX score was the factor most strongly associated with residual coronary disease. Each 1-point increase in baseline SYNTAX score was associated with approximately 1.93-fold higher odds of rSS positivity (OR, 1.925; 95% CI, 1.666–2.225; *p* < 0.001). Compared with NSTEMI, STEMI presentation was associated with lower odds of rSS positivity (OR, 0.169; 95% CI, 0.081–0.354; *p* < 0.001). In the reference model, age, sex, and admission glucose were not independently associated with the outcome (Table 2).

When evaluated separately as an addition to the reference model, HDL-C was inversely associated with rSS positivity (OR per 1-mg/dL increase: 0.965; 95% CI, 0.932–0.999; *p* = 0.045). Higher LDL-C/HDL-C and non-HDL-C/HDL-C ratios were positively associated with rSS positivity (OR per 1-unit increase, 1.337; 95% CI, 1.021–1.752; *p* = 0.035, and OR, 1.257; 95% CI, 1.017–1.553; *p* = 0.034, respectively). All variance inflation factors were below 1.30, indicating no evidence of problematic multicollinearity. Restricted cubic spline analyses showed no evidence of significant non-linearity in baseline SYNTAX score, age, admission glucose, HDL-C, LDL-C/HDL-C ratio, or non-HDL-C/HDL-C ratio.

Adding HDL-C, the LDL-C/HDL-C ratio, and the non-HDL-C/HDL-C ratio modestly improved model fit, as indicated by likelihood-ratio tests (*p* = 0.041, *p* = 0.034, and *p* = 0.031, respectively). However, the absolute reductions in AIC were small, ranging from 2.2 to 2.7 points, whereas BIC favored the more parsimonious reference model (Table 3).

**Figure 3:**
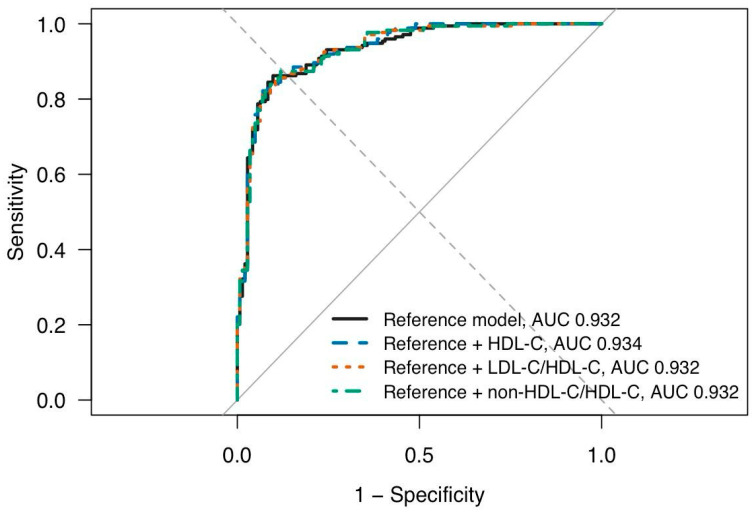
Comparative receiver operating characteristic curves of the reference and lipid-extended models for identifying residual coronary disease.

The reference model had an AUC of 0.932 (95% CI, 0.905–0.959). The corresponding AUCs were 0.934 (95% CI, 0.907–0.961) for the model including HDL-C, 0.932 (95% CI, 0.905–0.960) for the model including the LDL-C/HDL-C ratio, and 0.932 (95% CI, 0.905–0.960) for the model including the non-HDL-C/HDL-C ratio. None of the lipid-extended models significantly improved discrimination compared with the reference model in paired DeLong analyses (*p* = 0.463, *p* = 0.912, and *p* = 0.926, respectively; Table 3 and Figure 3).

Bootstrap internal validation using 1000 resamples yielded an optimism-corrected AUC of 0.926, a calibration intercept of −0.004, a calibration slope of 0.938, and a Brier score of 0.108 for the reference model. For the model including the non-HDL-C/HDL-C ratio, the corresponding values were 0.925, −0.001, 0.923, and 0.108, respectively. Thus, adding the non-HDL-C/HDL-C ratio did not improve optimism-corrected discrimination, calibration, or overall prediction error (Supplementary Table S1).

In models excluding the baseline SYNTAX score, associations with lipid parameters were stronger. HDL-C remained inversely associated with rSS positivity (OR per 1-mg/dL increase, 0.965; 95% CI, 0.941–0.988; *p* = 0.004), whereas the LDL-C/HDL-C ratio (OR per 1-unit increase, 1.329; 95% CI, 1.087–1.624; *p* = 0.005) and the non-HDL-C/HDL-C ratio (OR, 1.232; 95% CI, 1.057–1.436; *p* = 0.007) were positively associated with rSS positivity. The similarity of the effect estimates between models with and without the baseline SYNTAX score indicated that adjustment for initial anatomical disease burden did not materially alter the direction or magnitude of the lipid associations, although the strength of the statistical evidence was attenuated after this adjustment (Supplementary Table S2).

To reduce information loss associated with dichotomization, rSS was additionally categorized as 0, 1–8, and >8, comprising 143, 121, and 53 patients, respectively. Because the baseline SYNTAX score violated the proportional-odds assumption, partial proportional-odds models allowing its effect to vary across thresholds were used. In these ordinal analyses, higher HDL-C was associated with lower odds of belonging to a more severe rSS category (common OR per 1-mg/dL increase, 0.963; 95% CI, 0.935–0.992; *p* = 0.012). Higher LDL-C/HDL-C and non-HDL-C/HDL-C ratios were associated with higher rSS categories (common OR per 1-unit increase, 1.289; 95% CI, 1.036–1.604; *p* = 0.023, and OR, 1.205; 95% CI, 1.017–1.427; *p* = 0.031, respectively; Supplementary Table S3).

In expanded sensitivity models that were additionally adjusted for hypertension, diabetes mellitus, and left ventricular ejection fraction, the combined addition of these variables did not significantly improve the reference model (likelihood ratio *p* = 0.205). The directions and magnitudes of the lipid associations remained broadly similar; however, their confidence intervals included unity after the additional adjustment. The adjusted ORs were 0.967 (95% CI, 0.933–1.003; *p* = 0.069) for HDL-C, 1.273 (95% CI, 0.965–1.679; *p* = 0.087) for the LDL-C/HDL-C ratio, and 1.219 (95% CI, 0.983–1.511; *p* = 0.071) for the non-HDL-C/HDL-C ratio (Supplementary Table S4).

## 4. Discussion

In our study, we examined the relationship between lipid indices and residual coronary atherosclerotic burden (rSS) in patients presenting with ACS who underwent primary PCI. Because fasting lipid measurements were obtained during the acute phase of myocardial infarction, transient changes in lipid concentrations may have occurred despite blood sampling being performed within the first 24 h after admission. This should be considered when interpreting our findings. We found that conventional lipid parameters, including TC, TG, and LDL-C, were similar across groups. In contrast, low HDL-C levels and a high NHHR were significantly associated with a positive rSS. Furthermore, among the evaluated lipid indices, HDL-C and the non-HDL/HDL-C ratio showed the strongest independent associations with residual coronary disease in the multivariable logistic regression models.

The observed association between lower HDL-C levels and higher SYNTAX scores aligns with prior studies highlighting the protective effects of HDL in coronary artery disease (CAD). HDL particles promote reverse cholesterol transport, exert antioxidant and anti-inflammatory effects, and enhance endothelial function, collectively contributing to the stabilization or regression of atherosclerotic plaques [12,13,14]. Therefore, increasing serum HDL-C levels may reduce atherosclerosis progression and the risk of cardiovascular events [15]. In our cohort, despite similar LDL-C levels, HDL-C concentrations were lower in patients with positive rSS, suggesting that LDL-focused lipid management may be insufficient to reduce the risk of coronary events.

Non-HDL cholesterol is the sum of atherogenic cholesterol in the serum, and lowering it is an important target for controlling ATS [16]. Non-HDL cholesterol levels are strongly associated with the risk of atherosclerotic CVD [17]. A study found that high non-HDL-C levels independently predicted increased cardiovascular risk and were superior to LDL-C in predicting major cardiovascular events [4]. Sahin et al. reported that the syntax score in patients with premature coronary artery disease showed a strong positive correlation with non-HDL-C levels and a negative correlation with HDL-C levels [18]. A study reported that non-HDL-C had predictive value for acute myocardial infarction and sudden death in men with an LDL-C level < 120 mg/dL [19]. A recent intracoronary ultrasound study demonstrated a positive association between non-HDL-C levels (>130 mg/dL) and plaque burden and necrotic core volume. This also demonstrated an association between non-HDL-C levels and unstable plaques that cause acute coronary syndromes [20]. In our study, although non-HDL cholesterol levels were similar between the groups, the mean values exceeded the reference threshold of 130 mg/dL.

The NHHR and LDL/HDL-C ratio include both atherogenic and protective lipoproteins, providing a more comprehensive reflection of lipid balance and atherogenic burden than single lipid measures. Bhardwaj et al. found that TC and LDL-C levels were similar between the CAD and healthy control groups. However, HDL-C values were markedly lower in the patient group. Therefore, the LDL/HDL-C ratio helped identify individuals at risk for CVD [21]. The Helsinki Heart Study also showed that the LDL/HDL-C ratio better predicts CVD risk, especially in patients with high TG levels [22]. In a cohort of patients with premature CAD, Xu et al. reported that the LDL/HDL-C ratio was positively associated with disease severity and major adverse cardiovascular events over a 2-year follow-up period [23]. In a study of an elderly patient population, an elevated LDL/HDL ratio was associated with the severity of coronary artery disease. However, it did not emerge as an independent risk factor for predicting adverse cardiac or cerebrovascular events during the 2-year follow-up period [24]. Another study involving 2226 patients demonstrated that adverse cardiovascular and cerebrovascular events following PCI are closely associated with a high LDL/HDL-C ratio [25]. In our study, the mean LDL/HDL-C ratio was 3.5 [2.6–4.3] in the overall cohort with angiographically confirmed coronary artery disease, exceeding the commonly accepted reference threshold (>2.5). Additionally, the LDL/HDL-C ratio was significantly higher in the rSS-positive group and remained independently associated with residual SYNTAX score positivity in Model 3.

Early intervention in patients with high NHHR has been highlighted as a potentially beneficial strategy to reduce cardiovascular risk in this population [26]. In a study of patients with type 2 diabetes, with an average follow-up of 5.8 years, low NHHR was a better predictor of coronary heart disease risk than LDL-C, especially when NHHR was below 3 mmol/L [27]. Li et al. reported that NHHR, defined as the atherogenic index, predicts CAD severity [28]. Another study argued that the non-HDL-C/apolipoprotein A-I ratio, calculated using apolipoprotein A-I, the protein component of HDL-C, is associated with the severity of coronary artery lesions and can serve as a biomarker for assessing CAD [16]. Recent studies have highlighted the limitations of using LDL-C alone as a therapeutic target, particularly in ACS patients with high inflammatory and metabolic risks [29].

In our study, HDL-C was consistently and independently associated with residual SYNTAX score positivity across all regression models. Among the lipid indices evaluated, the non-HDL/HDL-C ratio showed the strongest independent association with residual SYNTAX score positivity in patients presenting with a first acute coronary syndrome.

Although the baseline SYNTAX score remained the strongest determinant of residual disease burden, the observed associations between lipid indices and residual SYNTAX score suggest that these readily available biomarkers may provide complementary information rather than serve as substitutes for established angiographic predictors.

## 5. Limitations

To our knowledge, this is the first study to conduct such an analysis. However, this study has several limitations. First, the single-center retrospective design may limit the generalizability of the results. Second, the number of participants enrolled in this study was relatively small. Third, the primary analysis dichotomized rSS as 0 versus >0 to distinguish complete from incomplete angiographic revascularization, potentially reducing information on the severity of residual disease. Nevertheless, the direction and statistical significance of the lipid associations were preserved in an ordinal sensitivity analysis using the established rSS categories of 0, 1–8, and >8, as well as in partial proportional-odds models. Fourth, although the primary models adjusted for relevant covariates, several potentially important variables, including body mass index, smoking status, standardized infarct localization, time from admission to angiography, and additional metabolic markers, were not consistently available for the entire cohort and therefore could not be included. Residual confounding, therefore, cannot be excluded. Finally, long-term adverse cardiovascular outcomes associated with the evaluated lipid indices were not examined, which may limit conclusions about their prognostic value.

## 6. Conclusions

Low HDL-C levels and elevated non-HDL/HDL-C and LDL-C/HDL-C ratios were associated with residual coronary disease in patients presenting with a first ACS and undergoing PCI. These associations were consistent across binary and ordinal analyses, supporting a relationship between lipid indices and increasing residual coronary burden. Because these indices are simple, inexpensive, and routinely available, they may provide useful complementary information about the metabolic background of patients with residual coronary artery disease.

## Figures and Tables

**Table 1:** Baseline Characteristics of Patients According to Residual SYNTAX Score Groups.

Variables	Overall *n* = 317	RSS (−) *n* = 143	RSS (+) *n* = 174	*p*
**Demographic and clinical parameters**				
Age (years), mean ± SD	60.64 ± 10.61	59.55 ± 10.82	61.54 ± 10.39	0.096
Male sex, *n* (%)	252 (79.5)	113 (79.0)	139 (79.9)	0.889
EF (%), median [IQR]	60 [50–60]	60 [50–60]	55 [50–60]	0.166
HT, *n* (%)	136 (42.9)	63 (44.1)	73 (42.0)	0.733
DM, *n* (%)	82 (25.9)	34 (23.8)	48 (27.6)	0.519
KOAH, *n* (%)	27 (8.5)	15 (10.5)	12 (6.9)	0.313
CVD, *n* (%)	22 (6.9)	7 (4.9)	15 (8.6)	0.267
STEMI vs. NONSTEMI	167 (52.7)	88 (61.5)	79 (45.4)	**0.005**
Syntax score, median [IQR]	8 [8–12]	6 [5–7]	12 [9–14]	**<0.001**
Residual syntax score, median [IQR]	2 [0–6]	0	6 [3–10]	**<0.001**
**Laboratory parameters**				
TC (mg/dL), median [IQR]	195 [169–222]	196 [167.5–219.5]	194.5 [169–222.8]	0.697
TG (mg/dL), median [IQR]	141 [104–197]	141 [104–200]	141.5 [105–193.5]	0.769
HDL (mg/dL), median [IQR]	38 [33–45]	39 [33–49]	37 [32.3–43.8]	**0.022**
LDL (mg/dL), median [IQR]	134 [110–153]	133 [105–151.5]	137 [112.3–153]	0.435
Rem-C (mg/dL), median [IQR]	19 [12–29]	19 [12–28]	20 [13–29.8]	0.444
Non-HDL C (mg/dL), median [IQR]	154 [124–182.5]	152 [122–181]	157 [131.2–184.4]	0.298
Non-HDL/ HDL ratio (mg/dL), median [IQR]	4 [3–5.1]	3.8 [2.7–4.9]	4.3 [3.3–5.2]	**0.030**
TG/HDL ratio (mg/dL), median [IQR]	3.6 [2.4–5.5]	3.4 [2.3–6]	3.7 [2.5–5.4]	0.336
LDL/HDL ratio (mg/dL), median [IQR]	3.5 [2.6–4.3]	3.3 [2.4–4.2]	3.6 [2.9–4.4]	**0.033**
Glucose (mg/dL), median [IQR]	117 [100–156]	114 [98–140.5]	124 [101.3–169.5]	**0.017**
Urea (mg/dL), median [IQR]	32 [26–40]	32 [26–38.5]	32 [26–41]	0.589
Cr (mg/dL), median [IQR]	0.92 [0.8–1.1]	0.93 [0.8–1.1]	0.91 [0.8–1.1]	0.415
AST (U/L), median [IQR]	24 [18–36]	23 [18–33]	25 [19–38.8]	0.132
ALT (U/L), median [IQR]	18 [14–27]	18 [14–27]	18 [14–27]	0.569
GGT (U/L), median [IQR]	24 [17–38]	23 [17–36.5]	26 [17–38]	0.206
CRP (mg/dL), median [IQR]	0.5 [0.3–0.9]	0.5 [0.2–0.9]	0.5 [0.3–1]	0.431
WBC (×10^3^/μL), median [IQR]	10.3 [8.5–12]	10.1 [8.8–12]	10.4 [8.4–12]	0.618
HGB (g/dL), median [IQR]	14.5 [13.3–15.6]	14.7 [13.5–15.7]	14.4 [13.2–15.5]	0.147
PLT (×10^3^/μL), median [IQR]	257 [218–303]	265 [224.5–305.5]	252 [215.3–301.8]	0.224
NEU (×10^3^/μL), median [IQR]	6.5 [5.1–8.5]	6.6 [4.9–8.5]	6.5 [5.1–8.5]	0.990
LYMPH (×10^3^/μL), median [IQR]	2.5 [1.7–3.4]	2.5 [1.8–3.5]	2.4 [1.7–3.2]	0.507
MON (×10^3^/μL), median [IQR]	0.6 [0.5–0.8]	0.6 [0.5–0.8]	0.6 [0.4–0.8]	0.870
MHR, (median [IQR])	1.6 [1.1–2.2]	1.5 [1.1–2.1]	1.7 [1.2–2.3]	0.293
NHR, (median [IQR])	17 [12.8, 22.1]	16.9 [12.2–21.8]	17.4 [13–22]	0.243
SII, (median (IQR))	674 (452–1080)	673 (428–1059)	676 (473–1068)	0.791

ACS: acute coronary syndrome; AST: aspartate aminotransferase; ALT: alanine aminotransferase; Cr: creatinine; CRP: C-reactive protein; CVD: cerebrovascular disease; DM: diabetes mellitus; EF: ejection fraction; GGT: gamma-glutamyl transferase; Hb: hemoglobin; HDL: high-density lipoprotein; HT: hypertension; LDL: low-density lipoprotein; LYM: lymphocytes; MHR: monocyte/HDL ratio; MON: monocytes; NEU: neutrophils; NHR: neutrophil/HDL ratio; NSTEMI: non-ST-elevation myocardial infarction; PLT: platelets; Rem-C: remnant cholesterol; RSS: residual SYNTAX score; SII: systemic immune-inflammation index; STEMI: ST-elevation myocardial infarction; TC: total cholesterol; TG: triglycerides; WBC: white blood cells.

**Table 2:** Multivariable logistic regression analyses for the presence of residual coronary disease.

Variable	Model 1: Reference OR (95% CI), *p*	Model 2: Reference + HDL-C OR (95% CI), *p*	Model 3: Reference + LDL-C/HDL-C OR (95% CI), *p*	Model 4: Reference + Non-HDL-C/HDL-C OR (95% CI), *p*
Baseline SYNTAX score, per 1-point increase	1.925 (1.666–2.225), **<0.001**	1.929 (1.665–2.235), **<0.001**	1.935 (1.669–2.243), **<0.001**	1.931 (1.666–2.237), **<0.001**
STEMI presentation (ref: NSTEMI)	0.169 (0.081–0.354), **<0.001**	0.160 (0.075–0.341), **<0.001**	0.170 (0.080–0.360), **<0.001**	0.171 (0.081–0.364), **<0.001**
Admission glucose, per 10-mg/dL increase	1.047 (0.994–1.102), 0.084	1.049 (0.997–1.105), 0.068	1.048 (0.995–1.104), 0.080	1.044 (0.991–1.100), 0.104
Age, per 1-year increase	0.993 (0.958–1.028), 0.677	0.999 (0.964–1.036), 0.955	1.003 (0.967–1.041), 0.872	1.005 (0.968–1.044), 0.782
Male sex (ref: female)	1.004 (0.425–2.374), 0.992	0.838 (0.350–2.007), 0.692	0.979 (0.412–2.325), 0.961	0.963 (0.405–2.289), 0.932
HDL-C, per 1-mg/dL increase	—	0.965 (0.932–0.999), 0.045	—	—
LDL-C/HDL-C ratio, per 1-unit increase	—	—	1.337 (1.021–1.752), **0.035**	—
Non-HDL-C/HDL-C ratio, per 1-unit increase	—	—	—	1.257 (1.017–1.553), **0.034**

The dependent variable was the presence of residual coronary disease, defined as a residual SYNTAX score > 0. The reference model included baseline SYNTAX score, ACS presentation, admission glucose concentration, age, and sex. HDL-C, LDL-C/HDL-C ratio, and non-HDL-C/HDL-C ratio were added separately to the reference model. OR: odds ratio; CI: confidence interval; HDL-C: high-density lipoprotein cholesterol; LDL-C: low-density lipoprotein cholesterol; NSTEMI: non-ST-elevation myocardial infarction; STEMI: ST-elevation myocardial infarction.

**Table 3:** Model fit, discrimination, and incremental value of lipid parameters.

Model	AIC	BIC	AUC (95% CI)	Likelihood-Ratio Test vs. Reference, *p*	DeLong Test vs. Reference, *p*
Reference model	230.2	**252.7**	0.932 (0.905–0.959)	—	—
Reference + HDL-C	228.0	254.3	0.934 (0.907–0.961)	0.041	0.463
Reference + LDL-C/HDL-C ratio	227.7	254.0	0.932 (0.905–0.960)	0.034	0.912
Reference + non-HDL-C/HDL-C ratio	**227.5**	253.9	0.932 (0.905–0.960)	0.031	0.926

The reference model included baseline SYNTAX score, ACS presentation, admission glucose concentration, age, and sex. Likelihood-ratio tests compared each lipid-extended model with the nested reference model. Paired DeLong tests compared the AUC of each lipid-extended model with that of the reference model. Lower AUC and BIC values indicate better model fit; however, the absolute AUC differences between the lipid-extended models were small, and BIC favored the more parsimonious reference model. AIC: Akaike information criterion; AUC: area under the receiver operating characteristic curve; BIC: Bayesian information criterion; CI: confidence interval.

## Data Availability

The data presented in this study are available on request from the corresponding author due to privacy reasons.

## Supplementary Materials

The following supporting information can be downloaded at: https://www.mdpi.com/article/10.3390/diagnostics16152412/s1, Table S1. Bootstrap internal validation of the reference and non-HDL-C/HDL-C models; Table S2. Sensitivity analyses excluding baseline SYNTAX score; Table S3. Ordinal sensitivity analyses according to residual SYNTAX score categories; Table S4. Expanded multivariable sensitivity analyses with additional clinical adjustment.

## Abbreviations

**SS**	Syntax Scores
**RSS**	Residual SYNTAX Scores
**HDL-C**	High-Density Lipoprotein Cholesterol
**LDL-C**	Low-Density Lipoprotein Cholesterol
**CVD**	Cardiovascular Disease
**ACS**	Acute Coronary Syndrome
**IHD**	Ischemic Heart Disease
**ATS**	Atherosclerosis
**HT**	Hypertension
**DM**	Diabetes Mellitus
**TG**	Triglycerides
**NHHR**	Non-HDL/HDL-C Ratios
**PCI**	Percutaneous Coronary Intervention
**STEMI**	ST-Elevation Myocardial Infarction
**NSTEMI**	Non-ST-Elevation Myocardial Infarction
**TC**	Total Cholesterol
**CAD**	Coronary Artery Disease
