# Supplementary material for: Comparative Analysis of Lipid Indices in Predicting Residual SYNTAX Score After Primary PCI in Patients with Acute Coronary Syndrome
Source: Diagnostics (Basel). 2026 Jul 31;16(15):2412. doi: 10.3390/diagnostics16152412 (PMC13465669; doi:10.3390/diagnostics16152412)
Supplement: Supplementary file 1 [file diagnostics-16-02412-s001.zip › Supplementary Tables S1-4.pdf]

Supplementary Table S1. Bootstrap internal validation of the reference and non-HDL-C/HDL-C models

Performance measure	Reference model	Reference model + non-HDL-C/HDL-C ratio
Apparent AUC	0.932	0.933
Optimism-corrected AUC	0.926	0.925
Optimism-corrected calibration intercept	−0.004	−0.001
Optimism-corrected calibration slope	0.938	0.923
Optimism-corrected maximum calibration error, Emax	0.054	0.056
Optimism-corrected Brier score	0.108	0.108
Internal validation was performed using 1,000 bootstrap resamples. The apparent and optimism-corrected AUCs were derived from Somers' Dxy using AUC = (Dxy + 1)/2. An ideal calibration intercept is 0, an ideal calibration slope is 1, and lower Emax and Brier scores indicate better performance. The reference model included baseline SYNTAX score, STEMI presentation, admission glucose concentration, age, and sex.		

Supplementary Table S2. Sensitivity analyses excluding baseline SYNTAX score

Lipid parameter	Adjusted OR (95% CI)	P value	Model AIC	Likelihood-ratio test vs clinical base model, P
Clinical base model	—	—	428.4	—
HDL-C, per 1-mg/dL increase	0.965 (0.941–0.988)	0.004	421.7	0.003
LDL-C/HDL-C ratio, per 1-unit increase	1.329 (1.087–1.624)	0.005	422.3	0.004
Non-HDL-C/HDL-C ratio, per 1-unit increase	1.232 (1.057–1.436)	0.007	422.9	0.006
The clinical base model included STEMI presentation, admission glucose concentration per 10-mg/dL increase, age, and sex but excluded baseline SYNTAX score. Each lipid parameter was added separately to the clinical base model. These analyses were performed to assess whether adjustment for initial anatomical disease burden materially influenced the associations between lipid parameters and residual coronary disease.				

Supplementary Table S3. Ordinal sensitivity analyses according to residual SYNTAX score categories

Lipid parameter	Adjusted common OR (95% CI)	p value	Likelihood-ratio test vs base model, p
HDL-C, per 1-mg/dL increase	0.963 (0.935–0.992)	0.012	0.011
LDL-C/HDL-C ratio, per 1-unit increase	1.289 (1.036–1.604)	0.023	0.023
Non-HDL-C/HDL-C ratio, per 1-unit increase	1.205 (1.017–1.427)	0.031	0.031

Residual SYNTAX score was categorized as 0, 1–8, and >8. Partial proportional-odds logistic regression was used because baseline SYNTAX score violated the proportional-odds assumption. The effect of baseline SYNTAX score was therefore allowed to vary across outcome thresholds, whereas proportional odds were retained for the remaining predictors. Each lipid parameter was evaluated in a separate model adjusted for baseline SYNTAX score, STEMI presentation, admission glucose per 10-mg/dL increase, age, and sex. An OR greater than 1 indicates higher odds of belonging to a more severe residual SYNTAX score category.

Supplementary Table S4. Expanded multivariable sensitivity analyses with additional clinical adjustment

Lipid parameter	Adjusted OR (95% CI)	P value	Likelihood-ratio test vs expanded base model, P
HDL-C, per 1-mg/dL increase	0.967 (0.933–1.003)	0.069	0.065
LDL-C/HDL-C ratio, per 1-unit increase	1.273 (0.965–1.679)	0.087	0.086
Non-HDL-C/HDL-C ratio, per 1-unit increase	1.219 (0.983–1.511)	0.071	0.068

The expanded base model included baseline SYNTAX score, STEMI presentation, admission glucose per 10-mg/dL increase, age, sex, hypertension, diabetes mellitus, and left ventricular ejection fraction. Each lipid parameter was added separately to the expanded base model. Although the direction and magnitude of the associations remained broadly consistent with the primary analyses, the confidence intervals included unity after additional adjustment.
